# A Protracted Course of COVID19 Infection in a Metastatic Breast Cancer Patient During CDK4/6 Inhibitor Therapy

**DOI:** 10.3389/fonc.2020.01085

**Published:** 2020-06-09

**Authors:** Albert Grinshpun, Irit Merlet, Hila Fruchtman, Dean Nachman

**Affiliations:** ^1^Sharett Institute of Oncology, Hadassah-Hebrew University Medical Center, Jerusalem, Israel; ^2^Radiology, Hadassah-Hebrew University Medical Center, Jerusalem, Israel; ^3^Internal Medicine A, Hadassah-Hebrew University Medical Center, Jerusalem, Israel

**Keywords:** COVID19, metastatic breast cancer, CDK4/6 inhibitors, pneumonia, palbociclib

## Abstract

We describe the first case report of a patient with COVID19 infection and metastatic breast cancer, while on systemic therapy with a CDK4/6 inhibitor. The patient had unique disease course, characterized with delayed symptomatology. The case highlights novel findings and stress careful and extended follow-up during COVID19 infection in patients taking biologic therapies affecting the immune system.

## Introduction

CDK4/6 inhibitors revolutionized the treatment of hormone receptor-positive, HER2-negative metastatic breast cancer (MBC). Recently, major publications have shown the clear advantage of these drugs (Palbociclib, Ribociclib, Abemaciclib) on outcomes of pre- and post-menopausal women with MBC (1). The impressive data and the relative safety of these agents made them widely popular first or second-line therapy for MBC (2). In numbers, the US market of CDK4/6 inhibitors is estimated in several billion dollars which translates to thousands of patients at a given moment in the US alone (3). Noteworthy, the main side effects of CDK4/6 inhibitors (mainly Palbociclib and Ribociclib) are neutropenia and leukopenia (more in Asians vs. non-Asians) (4). Although not leading to clinically significant susceptibility to complicated infections in major registration trials, there is a clear increase in respiratory infections during therapy (probably of viral etiology) (5).

Nowadays, the Severe Acute Respiratory Syndrome Coronavirus-2 (SARS-CoV-2 or COVID19) is an ongoing worldwide pandemic. Initial report notes that cancer patients might be more susceptible to severe infection (6). Furthermore, anti-cancer therapy 14 days before admission was associated with worse outcomes in this small cohort (*n* = 8) of Chinese cancer patients (6).

Herein, we describe the first patient hospitalized due to COVID19 infection while taking palbociclib for MBC.

## Case Report

We report of 49 years old female diagnosed with *de-novo* stage IV disease in 2019; ER/PR-positive, HER2 negative, grade 2 invasive ductal carcinoma of the breast with liver metastasis. The patient initiated monthly Goserelin, Letrozole 2.5 mg QD, and Palcociclib 125 mg QD on days 1–21, every 28 days. Recent systemic imaging with 18-FDG-PETCT in February 2020 showed only minimal pathologic uptake in the breast tumor (near-complete response to therapy). As required, the patient performed a complete blood count before each Palbociclib cycle; her average neutrophil and lymphocyte counts since Palbociclib initiation were 1.9 10E9/L and 2.19 10E9/L, in comparison to 4.43 10E9/L and 2.55 10E9/L during 7 years prior to cancer diagnosis, respectively (both statistically significant lower during Palbociclib administration, *p* < 0.05, *student's t-test, data not shown*). Additionally, the patient has multiple drug allergies to various antibiotics, positive family history for Lupus, and 4 years prior to her current presentation she underwent laparoscopic sleeve gastrectomy.

**Figure 1:**
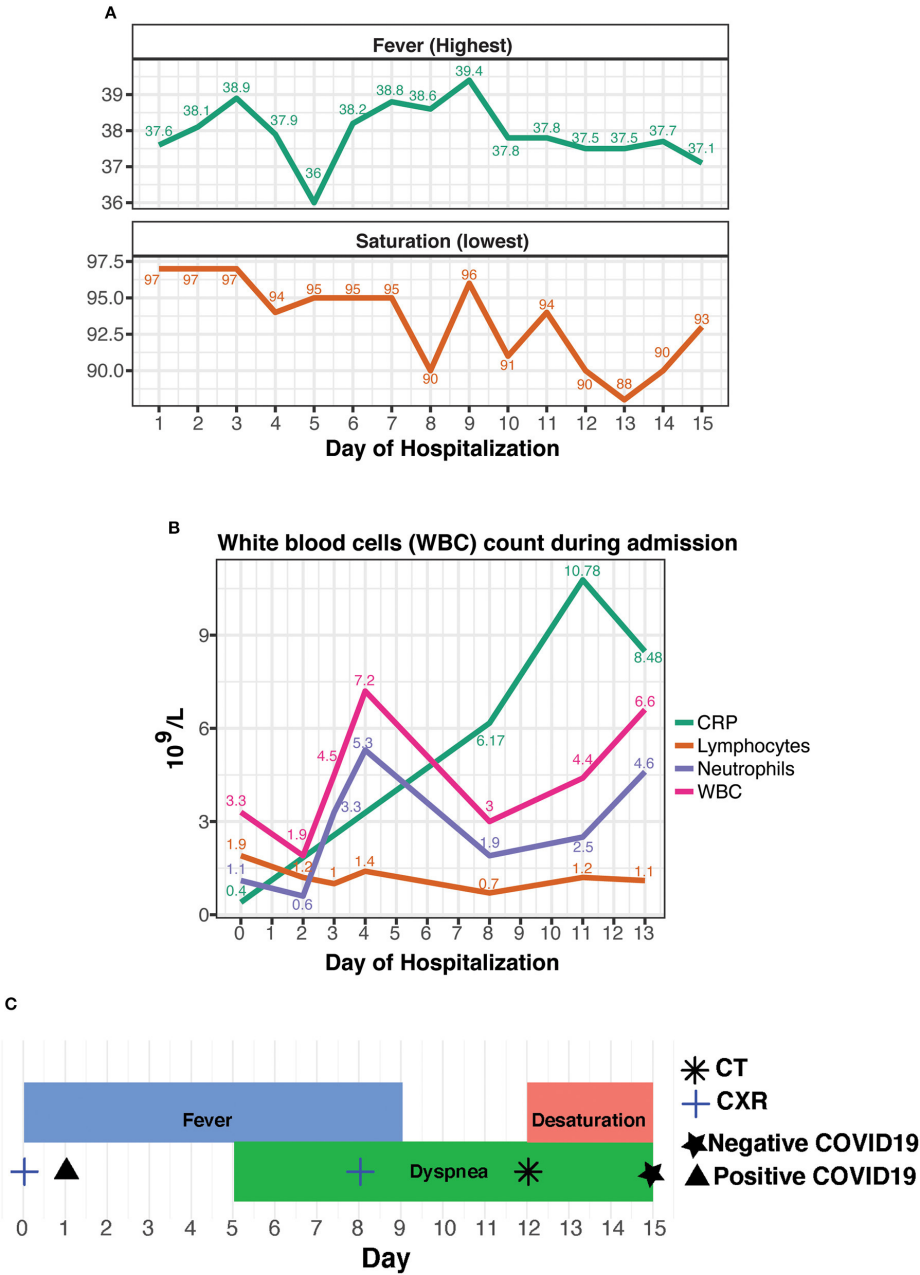
**(A)** Daily lowest non-invasive oximetry values in room air (as %) and daily highest fever (in °Celsius), as measured through hospitalization. **(B)** Leukocyte count and CRP results, as analyzed during admission. **(C)** A timeline of the hospitalization with concurrent events and tests.

During late March 2020, the patient was referred to the Emergency department (ED) with 4 days of fever, weakness, and nausea. On presentation to the ED (day 0), she was stable without dyspnea or fever, physical examination was impeccable. Blood tests for electrolytes, C-Reactive Peptide (CRP), liver, and kidney functions were all within normal limits. Blood count revealed mild leukopenia and moderate neutropenia of 1.1 10E9/L (Figure 1). A chest X-ray (CXR) was with no remarks, however, she was tested positive for COVID19 using real-time reverse transcription polymerase-chain-reaction (RT-PCR) from a nasopharyngeal swab. Due to the unique condition, the patient was hospitalized, and Palbociclib was held (day 1).

**Figure 2:**
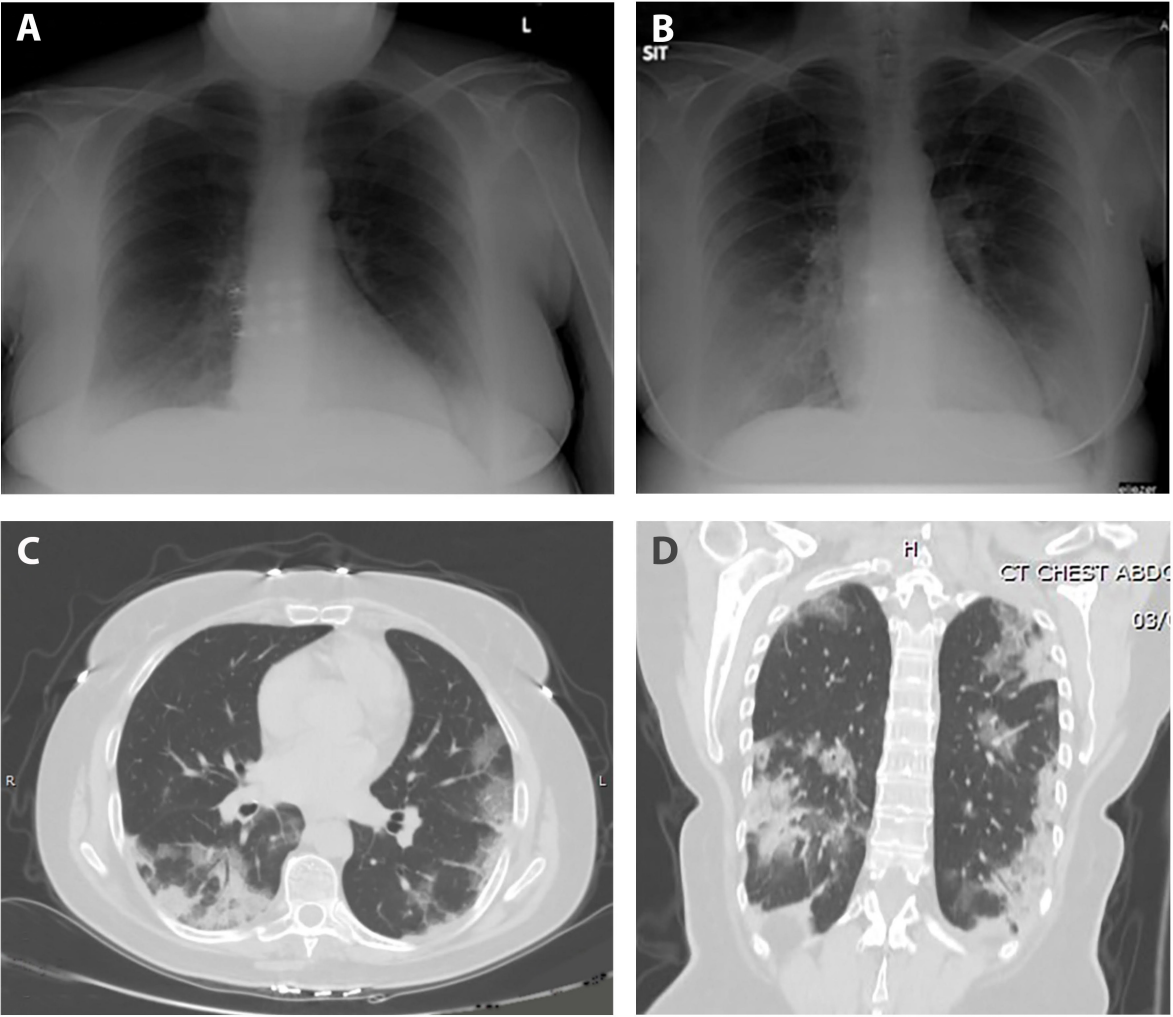
Anteroposterior chest radiograph; **(A)** normal on admission, **(B)** on day 8 of hospitalization with bilateral basal infiltrates. Axial **(C)** and coronal **(D)** sections CT scan; bilateral multifocal peripheral pulmonary ground glass opacities and consolidations typical for COVID19 infection.

During her hospital stay, she suffered from fevers of up to 39.4°C that persisted for 9 days, accompanied by worsening dyspnea that developed on the fifth day of her stay. Follow-up blood tests demonstrated normalization of the leukopenia and neutrophilia, alongside the occurrence of lymphopenia and elevated CRP. Due to the somewhat prolonged course of disease in a presumed immunosuppressed patient, further investigation was performed on 8th day of admission; CXR displayed new bilateral basal infiltrates, followed by non-contrast whole-body CT that was performed on day 11, showing bilateral pulmonary multifocal peripheral ground glass opacities and consolidations typical for COVID19 infection (Figure 2). Short term desaturation of as low as 88% on room air that resolved by oxygen delivery via a nasal cannula was observed on days 12–14 of her hospitalization. Subsequent to a gradual convalescence of symptoms during a 15 days hospitalization and two negative nasopharyngeal RT-PCR tests, the patient was discharged home in good general condition for further ambulatory medical care.

## Discussion

The main side effect of cdk4/6 inhibition therapy is a probable mild immunosuppression. A recent report highlights this issue by describing fulminant infection in a lymphopenic patient on CDK4/6 inhibitor therapy due to MBC (7). The current patient presented an unusually delayed course of the illness, as manifested by several key features. First, the patient demonstrated a delayed onset of clinical findings; dyspnea developed on the ninth day of the disease in comparison to a median of 5 to 7 days in previous cohorts of patients suffering from COVID19 (8, 9). True desaturation developed only on the 16th day of the disease (9–11). Second, when investigating blood tests, it is noticeable that lymphocyte count and CRP levels were normal on admission and became flawed only later in the course of the disease, unlike most patients who present with elevated CRP and lymphopenia (8, 10). Moreover, the median duration from onset of symptoms to imaging confirmation of pneumonia was 5 days in previous reports and 11 days with the current patient (11).

We hypothesize that Palbociclib administration prior to the hospital admission exerted a short-term immunosuppressive effect, halting the full presentation of the disease. This hypothesis is in line with another publication describing patients on immunosuppressive drugs for rheumatic disorders (12). Out of 4 patients with confirmed COVID19, only one needed supplementary low-flow oxygen for several days. Additional studies show that a key part of COVID19 pathogenesis is major recruitment of inflammatory cells into the lungs with a resulting cytokine storm (13). In our case, the rise of CRP levels linearly accompanied the clinical deterioration of the patient (Figure 1). Not surprisingly and as previously described (9–11), the fever preceded the respiratory symptoms. In contrary to CRP, the fever pattern itself or neutrophils' counts were fluctuant during disease development (due to the lymphopenia, the neutrophils were the major component of the white blood cell count). Therefore, we assume that once the drug was withdrawn, the full classic spectrum of illness appeared, including a bothering desaturation necessitating prolongation of hospital stay for close monitoring of the need for invasive ventilation. The noticed increase in neutrophils on days 2–4, strengthens our theory by probably representing the unleashed bone marrow's response to drug withdrawal.

The global COVID19 pandemic rushed several task forces, mainly dealing with inflammatory bowel disease, to issue recommendations and warnings regarding the usage of immunomodulatory medications (14). In light of the literature and the described patient, we suggest to the medical oncologists treating MBC patients with CDK4/6 inhibitors to weigh potential risks vs. expected benefit from this therapy.

Moreover, one may speculate that CDK4/6 inhibitors might serve as a repurposed immunomodulator for the treatment of patients with severe COVID19 infection. Yet, significant caution is needed when conclusions are drawn from this clinical vignette. Multiple genetic, immune and environmental factors (such as the expression ACE2 that binds to COVID19 virions) might have affected the clinical course of disease.

In conclusion, cancer patients are a potential risk group for severe COVID19 infection and therefore deserve vigilant surveillance these days (preferably distant). Additional careful data collection and monitoring of this group might shed light on novel approaches to attenuate the severity of illness in cancer patients and in the general population.

## Data Availability Statement

The original contributions presented in the study are included in the article/supplementary material, further inquiries can be directed to the corresponding author/s.

## Ethics Statement

A written informed consent was obtained from the patient for the publication of any potentially identifiable images/data included in this report.

## Author Contributions

All authors listed have made a substantial, direct and intellectual contribution to the work, and approved it for publication.

### Conflict of Interest

The authors declare that the research was conducted in the absence of any commercial or financial relationships that could be construed as a potential conflict of interest.
